# Holistic Optimization of Bioinformatic Analysis Pipeline for Detection and Quantification of 2′-O-Methylations in RNA by RiboMethSeq

**DOI:** 10.3389/fgene.2020.00038

**Published:** 2020-02-13

**Authors:** Florian Pichot, Virginie Marchand, Lilia Ayadi, Valérie Bourguignon-Igel, Mark Helm, Yuri Motorin

**Affiliations:** ^1^ IMoPA UMR7365 CNRS-UL, BioPole Université de Lorraine, Vandœuvre-lès-Nancy, France; ^2^ Epitranscriptomics and RNA Sequencing (EpiRNA-Seq) Core Facility, UMS2008 IBSLor (CNRS-UL)/US40 (INSERM), Université de Lorraine, Vandœuvre-lès-Nancy, France; ^3^ Institute of Pharmaceutical and Biomedical Sciences, Johannes Gutenberg-University Mainz, Mainz, Germany

**Keywords:** 2′-O-methylation, RNA, ribose methylation, high-throughput sequencing, bioinformatic pipeline, receiver operating characteristic

## Abstract

A major trend in the epitranscriptomics field over the last 5 years has been the high-throughput analysis of RNA modifications by a combination of specific chemical treatment(s), followed by library preparation and deep sequencing. Multiple protocols have been described for several important RNA modifications, such as 5-methylcytosine (m^5^C), pseudouridine (ψ), 1-methyladenosine (m^1^A), and 2′-O-methylation (Nm). One commonly used method is the alkaline cleavage-based RiboMethSeq protocol, where positions of reads' 5'-ends are used to distinguish nucleotides protected by ribose methylation. This method was successfully applied to detect and quantify Nm residues in various RNA species such as rRNA, tRNA, and snRNA. Such applications require adaptation of the initially published protocol(s), both at the wet bench and in the bioinformatics analysis. In this manuscript, we describe the optimization of RiboMethSeq bioinformatics at the level of initial read treatment, alignment to the reference sequence, counting the 5′- and 3′- ends, and calculation of the RiboMethSeq scores, allowing precise detection and quantification of the Nm-related signal. These improvements introduced in the original pipeline permit a more accurate detection of Nm candidates and a more precise quantification of Nm level variations. Applications of the improved RiboMethSeq treatment pipeline for different cellular RNA types are discussed.

## Introduction

The precise and high-throughput mapping of modified nucleotides in RNA is a real challenge in the field of epitranscriptomics (RNA modifications). Several recent publications have demonstrated the presence of numerous RNA modifications, not only in rather well studied species such as tRNA/rRNA/sn(sno)RNA, but also in coding RNAs (mRNA), in all living organisms studied to date. Thus, several high-throughput methods for the identification of RNA modifications have been developed and successfully applied for mapping m^5^C, m^6^A, pseudouridines, and (more recently) 2′-O-methylations (2′-O-Me), along with m^1^A and m^7^G/m^3^C/D (Dominissini et al., 2012; Squires et al., 2012; Carlile et al., 2014; Schwartz et al., 2014; Hauenschild et al., 2015; Dai et al., 2017; Marchand et al., 2018; Schwartz, 2018).

The major impediment to applying high-throughput screening methods resides in various experimental and bioinformatics biases, which are only partially controlled and may affect the precision of the final result or even lead to a nonnegligible number of false-positive identifications. Taking into account the size of eukaryotic transcriptomes, thousands of false-positive signals may appear at the transcriptome-wide level, even with extremely strict criteria for candidate site selection (e.g., False Discovery Rate [FDR]< 1%). Thus, every step in bioinformatic data treatment, conversion and manipulation should be optimized in order to minimize the number of potential false-positive signals.

Recently, we published a high-throughput deep sequencing-based approach, named RiboMethSeq, for mapping of 2′-O-methylations in highly abundant RNAs, mostly in rRNA (Marchand et al., 2016; Erales et al., 2017), with possible extension to tRNA (Marchand et al., 2017a; Freund et al., 2019). This protocol is also suitable for low abundance RNAs (Krogh et al., 2017). The RiboMethSeq protocol is based on the enhanced protection of the phosphodiester bond in RNA from nucleolytic attack and cleavage due to the presence of 2′-O-methylation at the 5′-neighboring ribose moiety. This enhanced protection is evaluated as a normalized number of 5′- and 3′-ends of randomly cleaved fragments present in the sequencing library. If a residue is 2′-O-methylated, this reduces the cleavage efficiency and thus the relative number of fragments starting and ending at +1 nucleotide relative to the modification. In the RiboMethSeq approach, such relative protection compared to neighbors is calculated using different scoring schemes, and the presence/absence of a 2′-O-methylation is then deduced on this basis. An alternative calculated score (ScoreC) also allows precisely measuring the methylation ratio at a given nucleotide.

In this work, we report the comprehensive optimization of every step of the bioinformatic treatment used for the detection and quantification of ribose 2′-O-methylation by the RiboMethSeq protocol. We systematically evaluated the importance and the impact of 5′- and 3′-trimming strategies, parameters for alignment to the reference sequence, as well as the use of specific calculated scores for 2′-O-Me mapping and quantification. Our results demonstrate that a reduced calculation interval is favorable for the general discrimination of 2′-O-Me signals from potential false-positive hits. We propose new, optimized scores (ScoreMEAN2, ScoreA2, and MethScore2) that provide better FDR values and also improve the relative quantification of 2′-O-methylation in RNA.

## Materials and Methods

### Biological Material

To optimize the RiboMethSeq scores, we used previously published datasets obtained for wild-type yeast *Saccharomyces cerevisiae* and human HeLa cell rRNA 2′-O-methylation analysis, as well as additional samples for hTERT immortalized human mammary epithelial cell line (HME) (Marchand et al., 2016; Erales et al., 2017; Sharma et al., 2017); accession numbers PRJEB43738, PRJEB34951 and PRJEB35565.

### RiboMethSeq Protocol

The RiboMethSeq protocol (Marchand et al., 2016; Marchand et al., 2017b) consists of random RNA fragmentation under alkaline conditions (96°C, pH 9.3, ~12−14 min for rRNA), an end-repair step consisting of de-phosphorylation of the 3′- ends of the RNA fragments and 5′-end phosphorylation, library preparation using 3′ -end and 5′-end ligation of adapters, an RT-step and PCR amplification coupled with barcoding. The resulting library is sequenced in paired-end PE 2x75 or, more commonly, in single-end mode (SR50) using an Illumina sequencing device (MiSeq or HiSeq1000).

### Trimming and Alignment

Adapter removal in this study was performed using the Trimmomatic utility (Bolger et al., 2014). With the default trimming parameters, the recognition of adapter sequences requires at least a 16 nt length. Shorter fragments of adapters are not recognized and thus are not removed. However, with a stringency of 7 (instead of 10), the adapter recognition requires only 10 nt. Considering this, 3′-end counting was carried out only for reads shorter than 40 nt after trimming. The alignment of raw reads was conducted by Bowtie2 (Langmead and Salzberg, 2012) in end-to-end mode.

### Comparing the Performance of 2′-O-Methylated Site Detection

For the selected datasets, we first applied the RiboMethSeq pipeline under standard conditions, and the previously described scores (ScoreMAX6, ScoreA, B and C) were calculated (Birkedal et al., 2015; Marchand et al., 2016). Score values were sorted in descending order, and the known modification status of every nucleotide (2′-O-methylated residue, pseudouridine, other modified residue or unmodified nucleotide) was attributed. Receiver operating characteristics (ROC) curves were plotted using these data, together with associated Matthews correlation coefficient (MCC) values. Other associated parameters of the ROC curves were calculated for maximal MCC value, taking into account true positive/false positive/true negative/false negative (TP/FP/TN/FN) hits. The performance of the treatment was evaluated on the basis of both the maximal MCC value and the associated FDR.

## Results and Discussion

### Brief Overview of RiboMethSeq Experiment

As described above, the high-throughput mapping of 2′-O-Me residues in RNA is based on random fragmentation of the phosphodiester bonds under mild alkaline conditions. The presence of a 2′-O-Me group protects the 3′-adjacent phosphodiester bond from nucleolytic cleavage, thus generating the characteristic gap in the 5′-end (as well as 3′-end) coverage profile of the sequencing library prepared from the fragmented RNA (**Supplementary Figure S1**). This enhanced protection is used as a signature for 2′-O-methylation and protection (and thus the gap's depth) and is supposed to be proportional to the level of 2′-O-Me at a given position.

In previously published studies (Birkedal et al., 2015; Marchand et al., 2016; Erales et al., 2017; Sharma et al., 2017), we and others used rather standard parameters for read trimming and alignment, and calculations arbitrarily used cleavage efficiency for 12 neighboring nucleotides (+/−6 from the methylation site). Scores allowing 2′-O-Me detection (ScoreMAX6 and ScoreA, called here ScoreA6), were calculated. The 2′-O-methylation level was assessed by calculating the MethScore (identical to the previously reported ScoreC, called ScoreC6 here).

### Selection of Representative Datasets for Optimization

**Figure 1:**
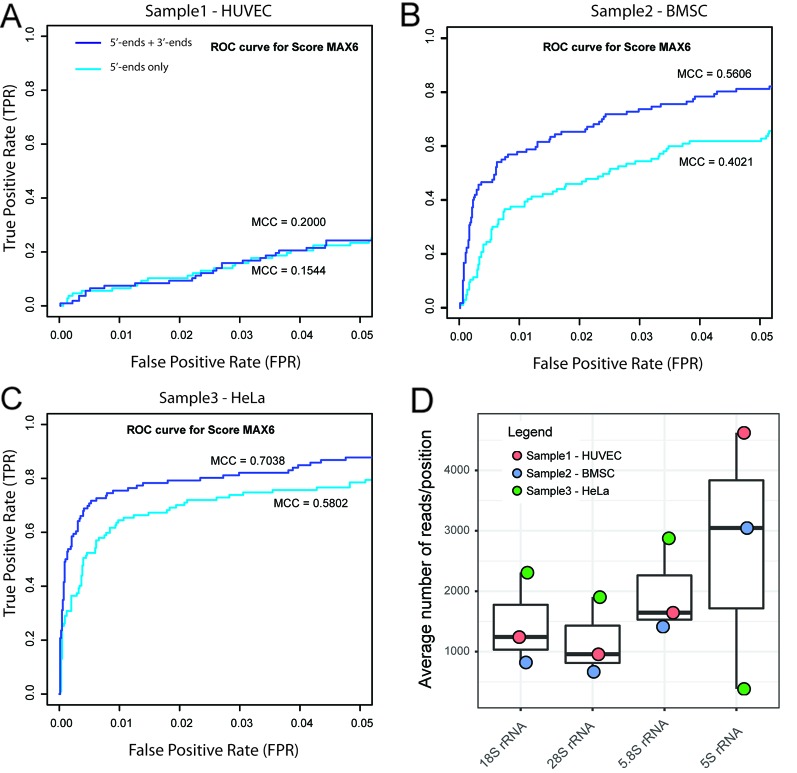
Selection of RiboMethSeq datasets for optimization. Three human datasets providing representative performance of 2'-O-Me detection (Sample 1 – HUVEC, 2 – BMSC, and 3 – HeLa) were selected on the basis of receiver operating characteristics (ROC) curves and the associated max Matthews correlation coefficient (MCC) values for ScoreMAX6 **(A**–**C)**. Graphs represent zoom to ROC curve 0–0.05 for false positive rate (FPR) and 0–1 for true positive rate (TPR). It was previously shown (Marchand et al., 2016) that 5'-end coverage (light blue curve) is sufficient for reliable construction of the RNA protection profile, but cumulated 5'- and 3'-end coverage (violet curve) provides better discrimination between methylated positions and false positive (FP) hits. **(D)** shows the read coverage per position for human rRNAs. 5S rRNA shows quite variable coverage, probably due to variations in 5S rRNA content in the total rRNA fraction due to biased extraction.

Initial screening and optimization of the RiboMethSeq bioinformatic pipeline was performed with >40 available human rRNA RiboMethSeq datasets obtained under standard, previously described (Marchand et al., 2016; Erales et al., 2017; Sharma et al., 2017), conditions of RNA fragmentation, sequencing, trimming, alignment and score calculation. We used a cumulative list of human modified rRNA positions reported in a 3D rRNA modification database (Piekna-Przybylska et al., 2008) and in the LBME snoRNA database (Lestrade and Weber, 2006), including two new positions that were recently reported (Krogh et al., 2016). Altogether, we considered 40 sites in 18S rRNA, 64 sites in 28S rRNA, and 2 positions in 5.8S rRNA (see **Supplementary Table S1**). Some of these positions are probably variably modified, or even not modified at all in some human cell lines or tissues (Krogh et al., 2016; Erales et al., 2017; Sharma et al., 2017); therefore, these incomplete modifications necessarily affect the number of FN hits and the max MCC values in the RiboMethSeq analysis. For each dataset, calculations of the RiboMethSeq scores were performed, and the performance of each dataset was evaluated for the detection of known rRNA 2′-O-methylated positions. Based on the preanalysis of available RiboMethSeq human rRNA samples, we selected three representative human datasets corresponding to two different cell lines (HUVEC and HeLa), as well as cultured bone marrow stem cells for further, more extensive analysis and optimization of the whole treatment pipeline (respectively named Sample 1 – HUVEC, 2 – BMSC, and 3 – HeLa (**Figure 1** and **Supplementary Figure S2**, **Supplementary Table S2**).

To allow a performance comparison between datasets, we selected samples with similar numbers of raw RiboMethSeq sequencing reads (>20 mln, see **Supplementary Table S2**).

### Minimal Number of Reads Required for Analysis

An optimized volume of sequencing reads required for complete RNA analysis is highly important, since it allows to obtain reliable results with a minimal sequencing cost per sample. From the analysis of the yeast rRNA samples (Marchand et al., 2016), sufficient coverage was evaluated to be ~ 750–1,000 reads/RNA position. For a more detailed analysis, we applied human rRNA datasets. Despite a similar number of raw sequencing reads (~20 mln), these datasets behaved differently regarding the precision of 2′-O-Me detection. Notably, the fraction of 5S rRNA reads varied substantially from one sample to another (**Figure 1**), probably also reflecting the different RNA extraction protocols used. However, there was no correlation between the total coverage and the prediction quality for 2′-O-Me. To define the minimal amount of raw sequencing information required for the successful application of RiboMethSeq analysis of human rRNA, we compared the performance of the method using a variable number of input reads for the same sample.

As anticipated, human 28S rRNA was the most difficult target to get full representation for all positions in the sequence. A comparison of missing positions in 28S rRNA in relation to the sequenced population is given in **Table 1**. A low number of raw reads (4 mln) can still be used, but numerous positions of 28S rRNA have zero raw read 5′-/3′-end counts in the final set. Despite this, the analysis of known 28S rRNA 2′-O-Me was not affected, because underrepresented regions are far away from these modified positions. Increasing the read number (8–12 mln, > 1,000 reads/nt) improves representativity, with only a marginal number of uncovered nucleotides, while 15–20 mln reads is recommended to achieve full coverage.

**Table 1:** Alignment statistics and uncovered rRNA positions in samples used for analysis.

Sample	number of raw reads used	4 mln	8 mln	12 mln	16 mln	20 mln
Sample 1 HUVEC	trimmed reads	3873996	7741257	11607806	15471803	19341955
	short reads for alignment	1761248	3581209	5338558	7104102	8909438
	aligned to rRNA reference	1513287	3077770	4587105	6104305	7657111
	uncovered pos 5S rRNA	0	0	0	0	0
	uncovered pos 5.8S rRNA	0	0	0	0	0
	uncovered pos 18S rRNA	**4**	**1**	0	0	0
	uncovered pos 28S rRNA	**100**	**37**	**21**	**11**	**7**
Sample 2 BMSC	trimmed reads	3878093	7752516	11628210	15494852	19371588
	short reads for alignment	1473330	2986750	4455805	5927702	7428697
	aligned to rRNA reference	999714	2027867	3023042	4022365	5042133
	uncovered pos 5S rRNA	0	0	0	0	0
	uncovered pos 5.8S rRNA	0	0	0	0	0
	uncovered pos 18S rRNA	**2**	**1**	0	0	0
	uncovered pos 28S rRNA	**18**	**5**	0	0	0
Sample 3 HeLa	trimmed reads	3882713	7764523	11644031	15516647	19387461
	short reads for alignment	2582027	5182220	7776132	10353556	12934621
	aligned to rRNA reference	2222928	4460722	6693085	8910528	11132036
	uncovered pos 5S rRNA	0	0	0	0	0
	uncovered pos 5.8S rRNA	0	0	0	0	0
	uncovered pos 18S rRNA	0	0	0	0	0
	uncovered pos 28S rRNA	0	0	0	0	0

The bold font highlights the number of uncovered positions in the different datasets.

### Minimal Trimming Length

The minimal trimming length used in the treatment pipeline may affect 2′-O-Me detection. Trimming parameters considerably influence the precision of 3′-end mapping for SR50 reads and the alignment quality. We thus tested variable minimal trimming lengths keeping alignment parameters constant. The calculated max MCC values for the tested human datasets showed no influence of these parameters on the final results, even if the number of ambiguously aligned short reads increased with a decreased minimal trimming length (**Supplementary Figure S3**). Depending on the length and complexity of the target RNA sequence, we recommend adapting the minimal length and Bowtie2 seed length (see below); the optimal seems to be 10 or 12 for human or yeast rRNA, or even lower for shorter RNAs (e.g., tRNA).

### Variation of Alignment Parameters

The original RiboMethSeq protocols (Marchand et al., 2016; Marchand et al., 2017b) used rather strict alignment parameters in Bowtie2: end-to-end mode, a minimal seed length of 22 nt and zero mismatches allowed in the seed (preset option “– sensitive”). The influence of the alignment mode (end-to-end versus local) was previously evaluated, and the soft read trimming performed in the local mode was found to be unsuitable for precise mapping of the read ends (Marchand et al., 2016). However, the seed length and number of mismatches allowed may also influence the quality of the alignment, since human rRNA has variations in nucleotide sequence and contains other modified nucleotides, which alter cDNA sequences, thus perturbing alignment to the reference.

To evaluate the importance of the alignment parameters, we proceeded with treatment using a reduced seed length and allowing (or not) mismatched nucleotides in the seed. The following parameter combinations were tested: seed lengths of 22 nt (default value for “– sensitive” preset option, and used previously), 16 nt, 12 nt, and 8 nt, allowing (or not) mismatched nucleotides in the seed.

The data in **Supplementary Figure S4A** show that the total proportion of aligned reads (unique or multiple alignments) vary only very slightly as a function of seed length and allowed mismatches; a seed length of 22 nt and mismatches in the seed allows only a slightly better alignment (69.41% vs. 65.41% of aligned reads). Variation of the Bowtie2 seed length does not much affect the max MCC value for both scores used for optimization (**Supplementary Figure S4B**). Similarly, allowing mismatches in the seed also has no influence on the final results. This shows that the alignment to the reference sequence is quite robust and mostly depends on the quality of sequencing data. Based on these observations, we recommend the use of 8–12 nt seed length, depending on the complexity and the length of the target RNA sequence. For better performance, the seed length can be coordinated with the minimal trimming size for sequencing reads (**Supplementary Figure S5**).

### Importance of the Calculation Window With Neighboring Nucleotides

**Figure 2:**
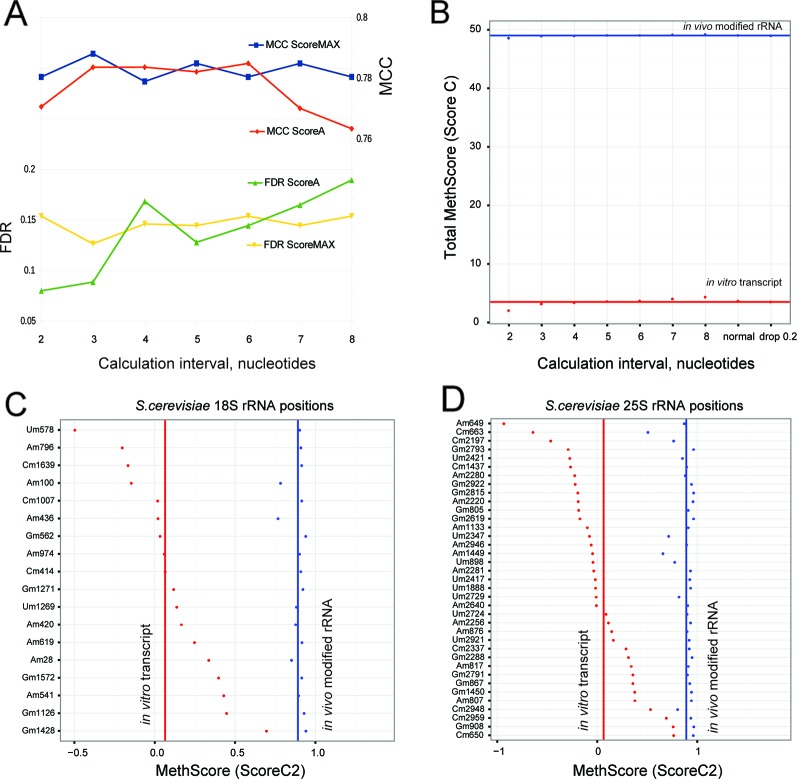
**(A)** Performance of ScoreMAX and ScoreA calculated using variable numbers of neighboring nucleotides (from +/−2 to +/−8). The standard RiboMethSeq protocol uses a +/−6 interval. Values for FDR and max Matthews correlation coefficient (MCC) are given. The scale on the left corresponds to false discovery rate (FDR), and on the right to MCC. Sample 2 – BMSC was used here for all calculations; other datasets gave similar trends. **(B)** shows global values for MethScore (ScoreC) calculated for modified yeast rRNA and *in vitro* rRNA transcripts using different neighboring intervals. The total number of “2'-O-Me groups” in rRNA is given (red - *in vitro* transcript, blue - modified rRNA). **(C**, **D)** MethScores2 (ScoreC2) for individual 2'-O-methylated positions in 18S **(C)** and 25S rRNA [**(D)**, red - *in vitro* transcript, blue - modified rRNA]. Lines correspond to average values.

In the originally published RiboMethSeq protocols (Birkedal et al., 2015; Marchand et al., 2016), 12 neighboring nucleotides (+/−6 nt window) were taken into account to calculate the 2′-O-Me scores (ScoreA, B and C). Since the size of this window was arbitrarily selected, we explored the influence of the window's size on the discrimination of 2′-O-Me signals from background. We compared the maximal MCC and the FDR values for the calculation interval from +/−2 nt up to +/−8 nt (window of 4 to 16 nt). The graph in **Figure 2A** shows that the ScoreMAX is nearly insensitive to the size of the calculation window, while ScoreA shows the best performance (and the lowest FDR) with the smallest window size (+/−2 nt). A larger window size has a detrimental effect for both scores. On the basis of these observations we suggest reducing the calculation window size to four neighboring nucleotides (Score 2 calculation scheme, +/−2 nt); the scores calculated with this window are referred to as ScoreMAX2 and ScoreA2.

### Quantification of 2′-O-Methylation With MethScore (ScoreC)

In the original RiboMethSeq protocol (Marchand et al., 2016), the MethScore [identical to ScoreC (Birkedal et al., 2015; Krogh et al., 2016)] was used for quantification of the 2′-O-Me level because the MethScore demonstrates a linear dependence on the depth of the gap in a cleavage profile, which is supposed to represent the protection and thus the degree of 2′-O-Me. In an ideal situation of a homogeneous cleavage profile, the MethScore varies from 0 to 1.0 and thus can be used as a measure of the degree of RNA methylation. In the case of yeast rRNA studied previously, the MethScore varied from negative values to 1.0, while conserving the linear dependence on the methylation rate (Marchand et al., 2016).

We noticed that RiboMethSeq detection scores behave better with a reduced calculation interval (+/−2 nt); therefore, we also explored variations of the MethScore. To select the best calculation interval, we compared the MethScore for intervals from two to eight neighboring nucleotides for naturally modified yeast rRNA and for unmodified synthetic rRNA transcripts. The MethScore values were expected to reach maximum for naturally modified rRNA and minimum for the unmodified counterpart. **Figure 2B** shows the difference between the MethScore values calculated for variable intervals for rRNA and synthetic transcript. For MethScore6 (+/−6 nt), we also tested different weight contributions of neighboring nucleotides.

The MethScore demonstrates the maximal cumulative difference between rRNA and synthetic transcript for the shortest interval of +/−2 nt; the other tested intervals gave roughly the same results. On the basis of this observation, we suggest calculating the MethScore for two neighboring nucleotides (referred to as MethScore2).

A detailed analysis, position by position, for 18S and 25S rRNA (**Figures 2C, D**) shows that methylated yeast rRNA displays MethScore2 values close to 0.9–1.0 for almost all modified positions (blue dots and line for average value), while the average level for synthetic unmodified transcript (red dots and line) is rather low. However, it is notable that the MethScore2 values for unmodified transcript are extremely variable, ranging from −1 to almost 0.9. For a limited subset of sites (Gm1428 in SSU 18S-rRNA and four positions in LSU 25S-rRNA), the difference of MethScore2 between modified and unmodified RNA is as low as 0.1. Precise measuring of the 2′-O-Me level variations at these rRNA positions is thus extremely difficult. However, over 90% of methylation sites display considerable MethScore2 differences between the modified and unmodified state, thus validating relative quantification of the methylation rate. Absolute values of the 2′-O-methylation cannot be directly measured using the RiboMethSeq approach; however, for a limited subset of sites the values of MethScore2 are comparable with independent measurements of 2′-O-Me rates assessed by LC-MS/MS (Buchhaupt et al., 2014; Taoka et al., 2016).

### Optimization of the ScoreMAX

The originally used ScoreMAX6 (Marchand et al., 2016) was designed to favor directional 5'- > 3′ gap depth compared to the opposite orientation. This design was based on the assumption that directional 5'- > 3′ drop is more informative for measuring the protection of the phosphodiester bond, while the drop in the opposite direction (3′- > 5') may represent nonspecific RNA structural effects. To verify the validity of such assumption, we tested different variants of ScoreMAX and their performance in the detection of 2′-O-Me. We compared the ScoreMAX2 (+/−2 nt window) with two modified versions. The first modified score retained the maximal value of normalized 5'- > 3′ and 3′- > 5' drop (ScoreMAX-MAX), while the second version calculated the average value between the two (ScoreMEAN2).

Calculation of the max MCC value demonstrated that ScoreMAX-MAX is less performant than the original ScoreMAX2, while ScoreMEAN2 shows better discrimination of FP hits. For the same dataset, Sample 3 – HeLa application of the ScoreMEAN2 allows attainment of the maximal MCC value of 0.7954 and reduces the FDR from 27% to 4%. A similar tendency was observed for two other datasets (**Supplementary Table S3**).

An important source of FP hits in RiboMethSeq analysis is the reduced ligation efficiency observed when modified RNA nucleotides are present at the 5'-end extremity of the fragment. This was experimentally observed for pseudouridine and other rare RNA modifications (Birkedal et al., 2015; Marchand et al., 2016). Since this reduced ligation efficiency also generates a “gap” in the sequencing profile, such FP signals are difficult to discriminate from undermethylated 2′-O-Me sites. Both scores (MEAN2 and A2) show a fair separation of values for 2′-O-Me nucleotides (blue) and unmodified residues (gray), but the peak for pseudouridines (red) partially overlaps with that of 2′-O-methylation. ScoreA2 shows better separation of 2′-O-Me and pseudouridine signals, while ScoreMEAN2 demonstrates a higher MCC and thus better performance for 2′-O-methylation detection (**Supplementary Figure S6**).

We also attempted to cumulate values for ScoreA2 and ScoreMEAN2 together by calculating their normalized sum (ScoreD). Despite the fact that ScoreA2 and ScoreMEAN2 generally pick out different FP hits, ScoreD does not further improve the performance (max MCC and FDR) compared to ScoreMEAN2 alone. In conclusion, the best results for detecting 2′-O-methylated residues were obtained with a calculation window of 4 nt (+/−2 nt) using ScoreA2 and ScoreMEAN2.

### Validation of ScoreMEAN2 and ScoreA2 With Human and *S. cerevisiae* rRNA Datasets

**Figure 3:**
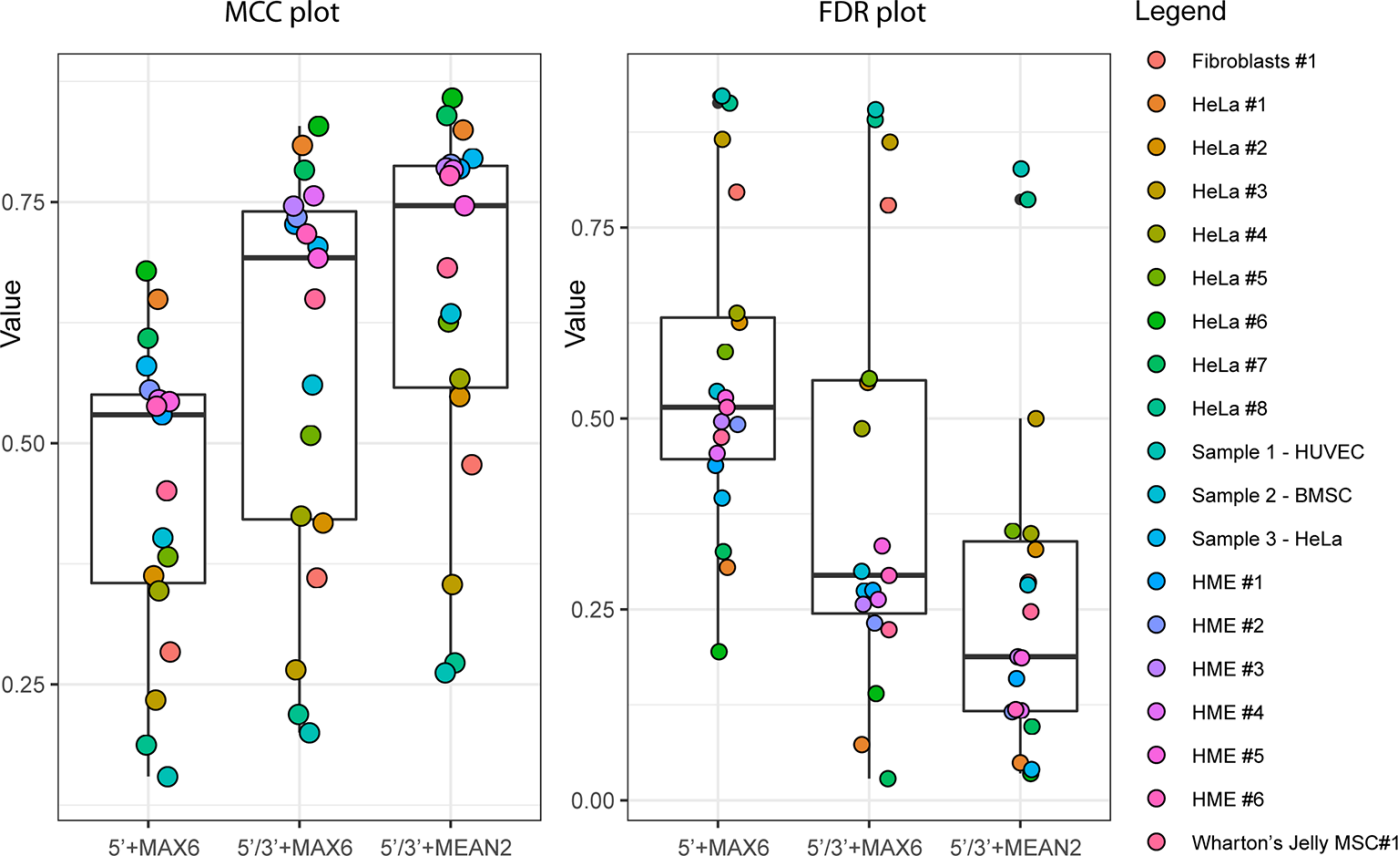
Improvement of ScoreMAX/MEAN (MAX6 and MEAN2) with 5'/3'-counts and reduced calculation window (Score 2 calculation scheme). Boxplot shows max Matthews correlation coefficient (MCC) values (left) and associated false discovery rate (FDR) (right) for all 19 RiboMethSeq datasets used for validation. Identity of the RiboMethSeq datasets is given on the right.

**Figure 4:**
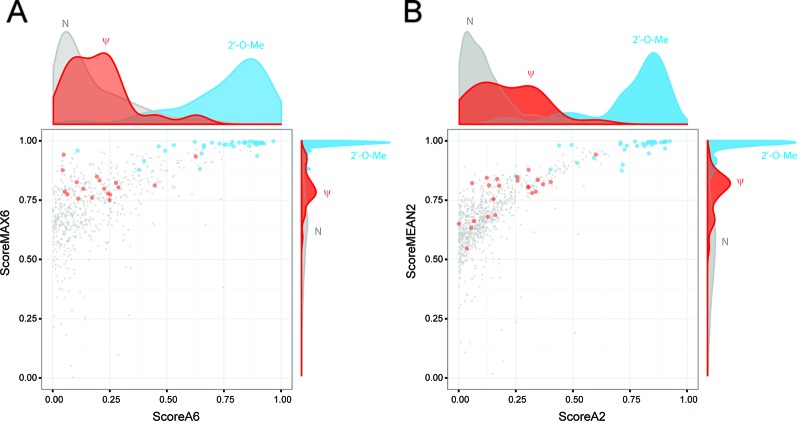
Validation of ScoreMEAN2 and ScoreA2 with the *S. cerevisiae* rRNA RiboMethSeq dataset. Comparative distribution of ScoreA6/ScoreMAX6 signals **(A)** and ScoreA2/ScoreMEAN2 signals **(B)** for the same *S. cerevisiae* rRNA dataset. Graphs represent scatter plots for two scores, with the associated density plot on top (ScoreA6 or ScoreA2) and on the right (ScoreMAX6 and ScoreMEAN2). RiboMethSeq signals for 2'-O-Me positions (light blue), pseudouridines (red) and unmodified nucleotides (gray) are shown.

To compare improvements associated with the use of ScoreMEAN2 and ScoreA2, we used independent human RiboMethSeq datasets obtained for other HeLa samples, human mammary epithelial cells (HME), human fibroblasts and Wharton's jelly MSC (19 altogether). Comparison of max MCC and FDR values obtained for Scores MAX6/MEAN2 and Scores A6/A2 calculated with different scoring schemes is shown on **Figure 3** and **Supplementary Figure S7**). In all cases, calculation scheme Score 2 improved detection of rRNA 2′-O-methylation (**Supplementary Table S3**). Additional validation was also performed with yeast *S. cerevisiae* rRNA RiboMethSeq dataset. **Figures 4A, B** show a side-by-side comparison of ScoreMAX6 and ScoreA6 values with their respective distributions (panel A), as well as ScoreMEAN2 with ScoreA2 (panel B). The new scoring scheme provides better separation of 2′-O-Me signals from those for pseudouridine and unmodified nucleotides, which is also confirmed by a better FDR and MCC values (**Supplementary Figure S8**). These data validate the newly proposed ScoreMEAN2 and ScoreA2, which can now be used for the detection of 2′-O-methylation in various RNA types.

## Conclusion

The results of this optimization suggest that RiboMethSeq analysis of rRNA can be performed using short trimming lengths (10–12 nt) and adapted Bowtie2 alignment parameters, which allows a gain in sequencing information. Calculations of RiboMethSeq scores for short calculation intervals (4 neighboring nucleotides) and ScoreMEAN2 and ScoreA2 can be used for mapping of the modified positions. Quantification of 2′-O-Me is accomplished using MethScore2, also calculated for four neighbors. A minimal of 4–6 mln of raw reads (~400 reads/nt on average) can be used to evaluate the methylation level for known rRNA methylation sites, but at least 15 mln reads (~1,500 reads/nt on average) should be used to discover new methylation candidates. For different RNA species such as tRNAs, the trimming/seed length can be further reduced (up to 8 nt), but the calculation of RiboMethSeq scores with four neighbors can be maintained. The optimal read coverage is somewhat similar (15–20 mln raw reads for yeast/human tRNAs, respectively).

## Data Availability Statement

We used previously published datasets obtained for wild type yeast *S. cerevisiae* and human HeLa cells rRNA 2′-O-methylation analysis as well additional samples (Marchand et al., 2016; Erales et al., 2017; Sharma et al., 2017), accession numbers PRJEB43738, PRJEB35565 and PRJEB34951.

## Author Contributions

FP—designed and optimized treatment pipeline. VM, LA and VB-I—performed RiboMethSeq library preparation and analysis. VM, MH and YM—wrote the manuscript.

## Conflict of Interest

The authors declare that the research was conducted in the absence of any commercial or financial relationships that could be construed as a potential conflict of interest.
