# Supplementary material for: Holistic Optimization of Bioinformatic Analysis Pipeline for Detection and Quantification of 2′-O-Methylations in RNA by RiboMethSeq
Source: Front Genet. 2020 Feb 13;11:38. doi: 10.3389/fgene.2020.00038 (PMC7031861; doi:10.3389/fgene.2020.00038)
Supplement: Supplementary file 1 [file Presentation_1.pdf]

Holistic optimization of bioinformatic analysis pipeline for detection and quantification of 2'-O-methylations in RNAs by RiboMethSeq

Florian Pichot[1,2], Virginie Marchand[1,2], Lilia Ayadi[1,2], Valérie Bourguignon-Igel[1,2], Mark Helm[3], Yuri Motorin[1,2*]

Supplementary Figures and Tables

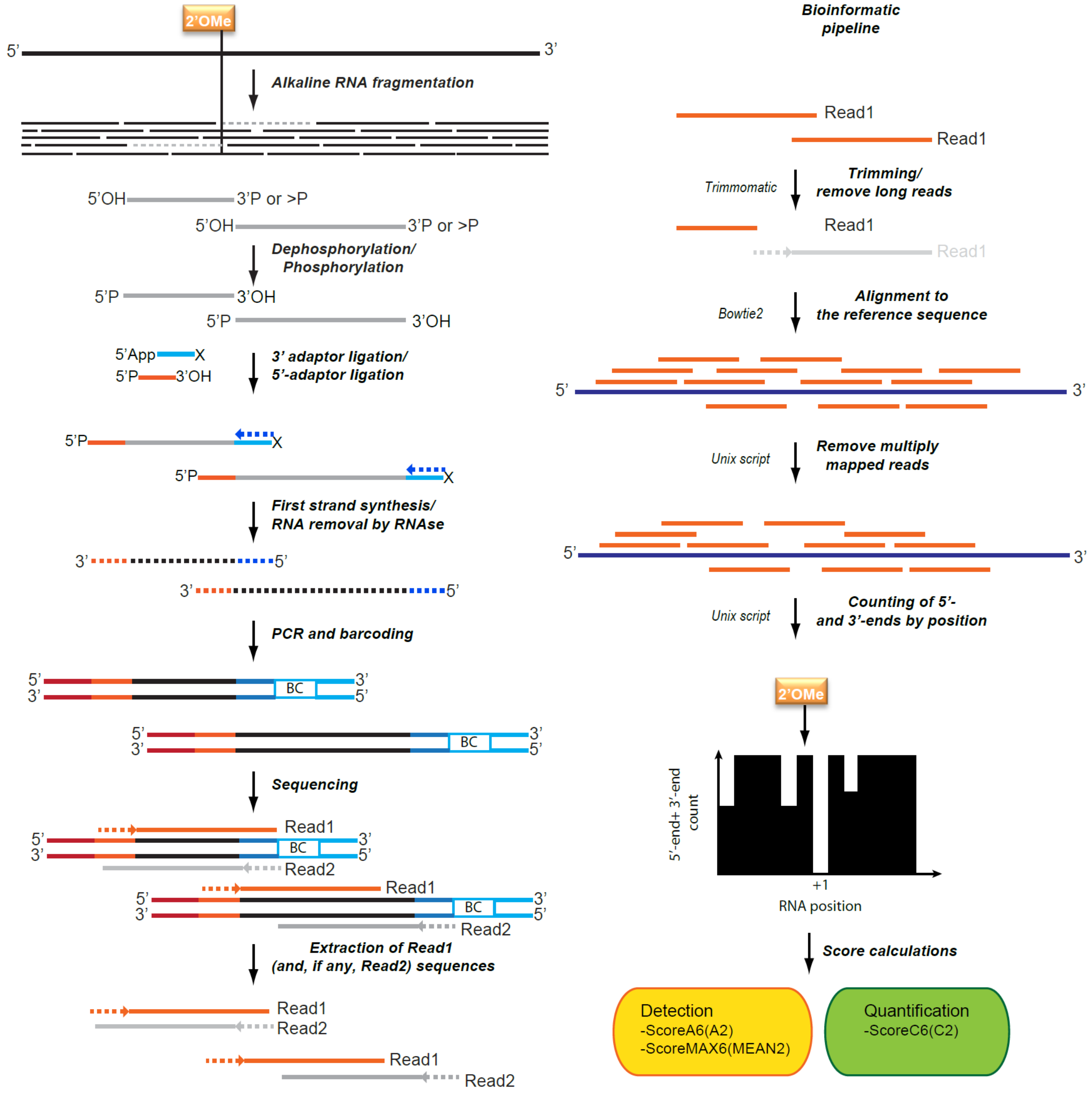

Figure S1 Outline of RiboMethSeq protocol for 2'-O-methylation detection in RNAs, "wet" lab steps and major steps of the data treatment pipeline are shown.

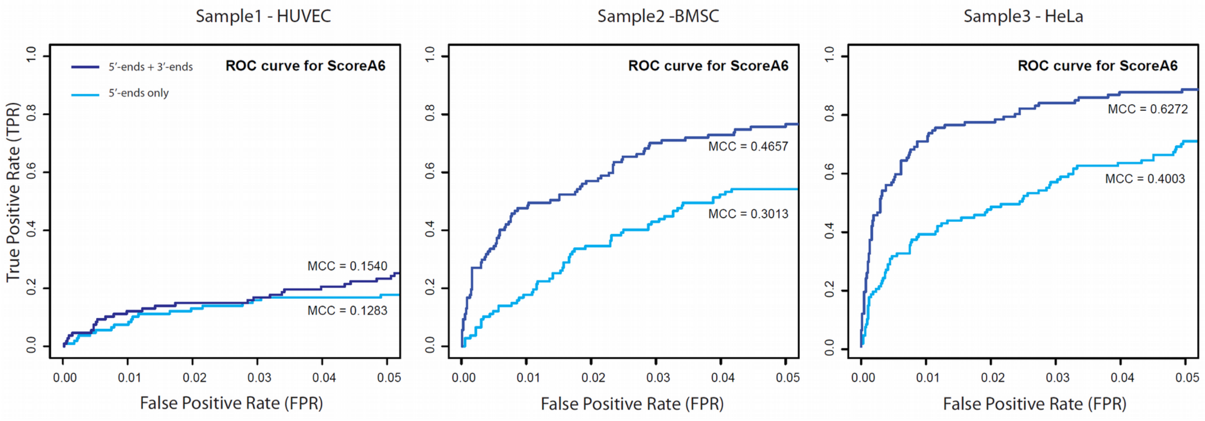

Figure S2 Selection of RiboMethSeq datasets for optimization. Three datasets providing representative performance in 2’-O-Me detection (Sample1 - HUVEC, 2 - BMSC, and 3 -HeLa, from left to right) were selected on the basis of ROC curve and associated max MCC values for ScoreA6. Graphs represent zoom to ROC curve 0-0.05 for FPR, 0-1 for TPR. It was previously shown that 5’-end coverage only (light blue curve) is sufficient for reliable construction of RNA protection/2’-O-Me profile, but cumulated 5’- and 3’-ends coverage (dark blue curve) generally provides better discrimination between methylated positions and FP hits.

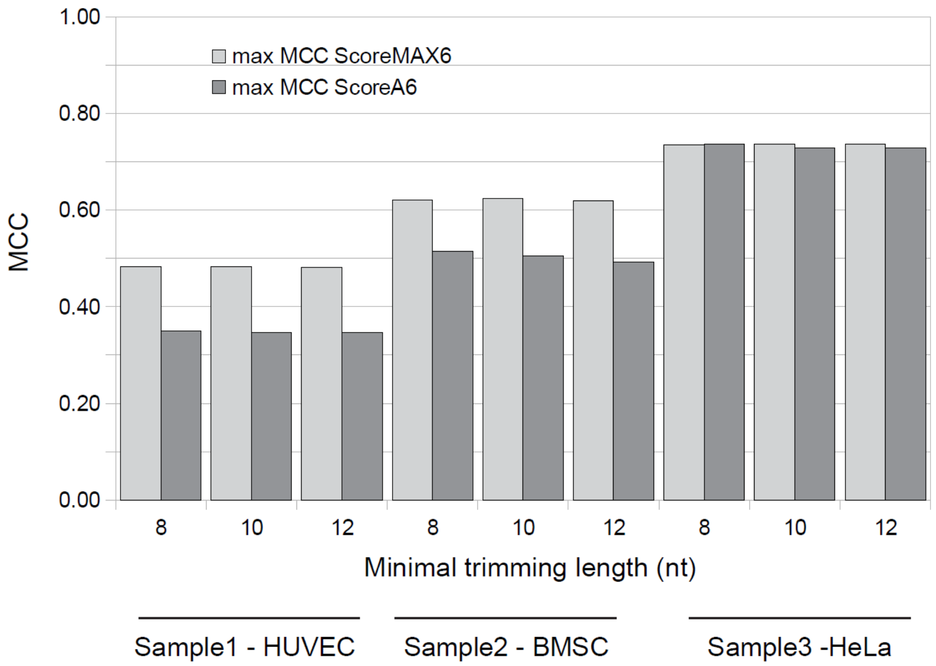

Figure S3 Influence of trimming parameters on max MCC values for ScoreMAX (MAX6) and ScoreA6. Trimming of raw reads was done using three minimal length (8 nt, 10 nt and 12 nt). Barplot shows max MCC values for ScoreMAX6 and ScoreA6, for three samples (Sample1 - HUVEC, 2 -BMSC and 3 - HeLa) retained for evaluation.

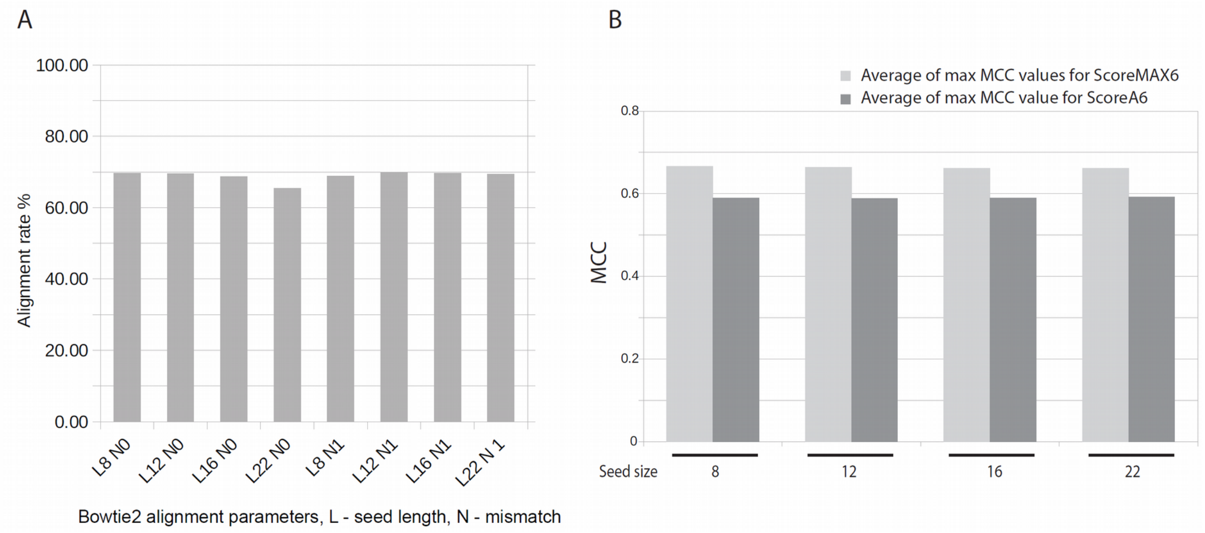

Figure S4 Influence of the Bowtie2 alignment parameters on the proportion of total aligned reads and on the performance of 2’-O-Me detection. Alignment statistics for Sample2- BMSC with different seed length and mismatch (Panel A). Panel B - ScoreMAX (MAX6) gives better performance compared to ScoreA6 in detection of 2’-O-Me sites, at all seed length tested. Average values of max MCC calculated for Samples1 - HUVEC, 2 - BMSC and 3 - HeLa.

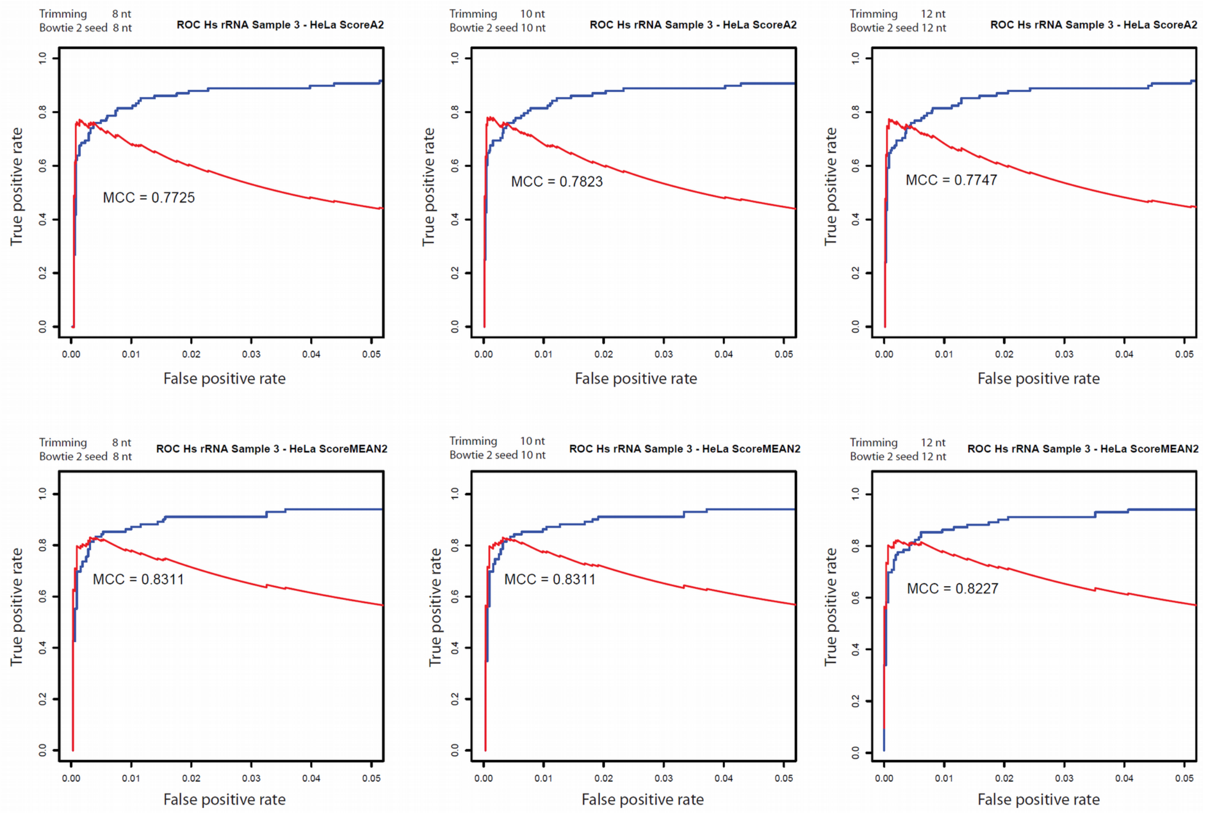

Figure S5 Measured performance for combinations Trimming/Bowtie2 parameters (trimming length/Bowtie2 seed length) for ScoreA2 and ScoreMEAN2. Sample 3 - HeLa was used for calculations of ROC curves. Blue curve – TP hits, red curve- MCC. Max MCC value is given on the graph. The same coordinates as in Figure S1 were used (FPR 0-0.05, TPR 0-1).

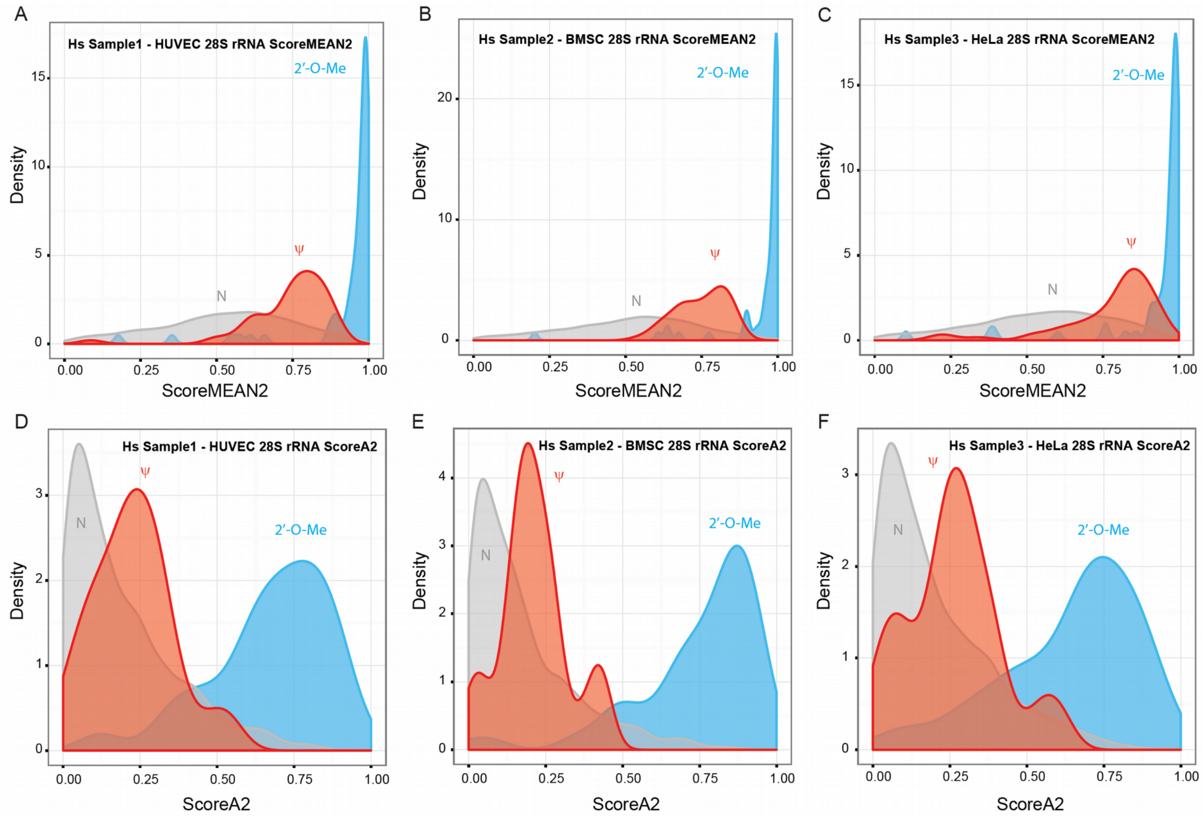

Figure S6 Density plots for signals observed at 2'-O-Me positions (light blue), pseudouridines (red) and unmodified nucleotides (gray) in human 28S rRNA. Graphs were made for Sample1 – HUVEC, 2 – BMSC, and 3 – HeLa. Samples1, 2 and 3 (Panels A/D, B/E and C/F, respectively, and for ScoreMEAN2 and ScoreA2 (top and bottom, respectively).

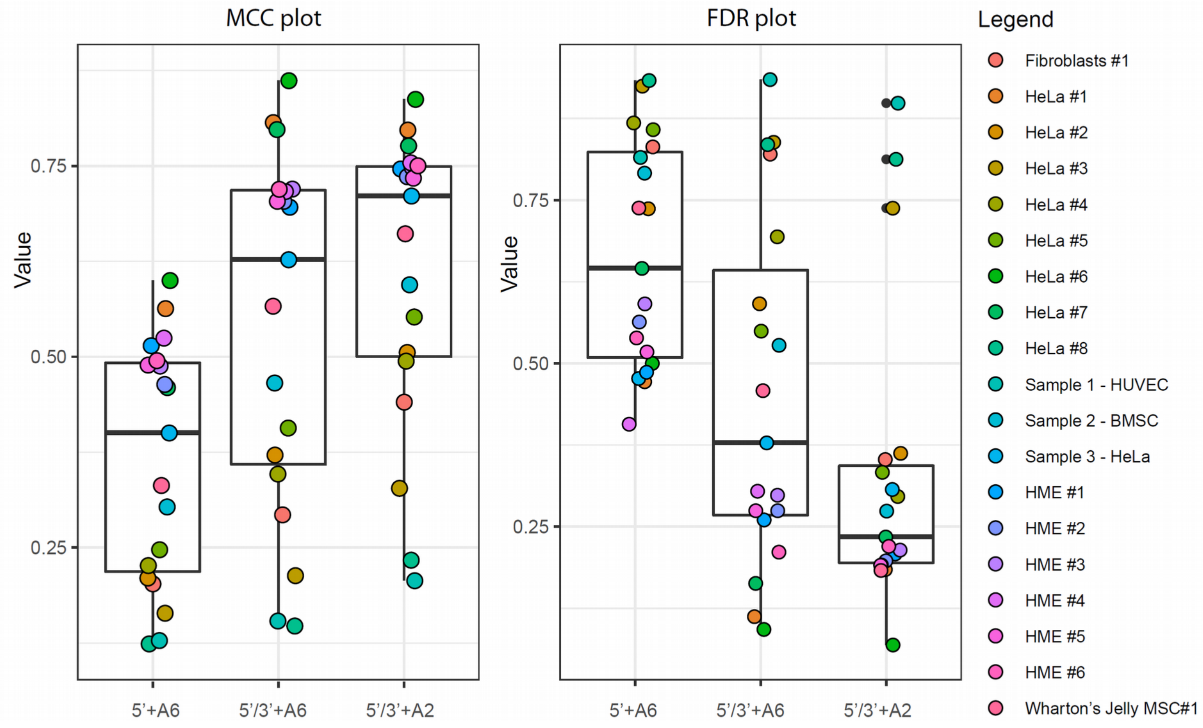

Figure S7 Improvement of ScoreA (A6 and A2) with 5'/3'-counts and reduced calculation window (Score 2 calculation scheme). Boxplot shows max MCC values (left) and associated FDR (right) for all 19 RiboMethSeq datasets used for validation. Identity of the RiboMethSeq datasets is given on the right.

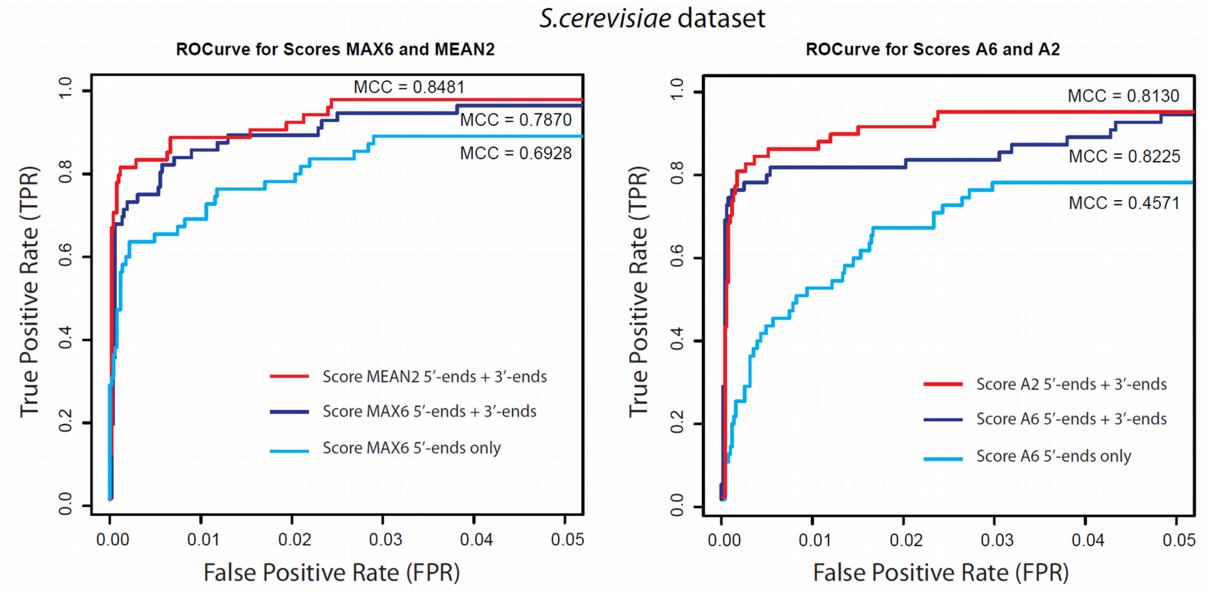

Figure S8 Validation of RiboMethSeq Score 2 calculation scheme using S.cerevisiae rRNA dataset. Graphs represent zoom to ROC curve 0-0.05 for FPR, 0-1 for TPR. It was previously shown that 5'-end coverage only (light blue curve) is sufficient for reliable construction of RNA protection/2'-O-Me profile, but cumulated 5'- and 3'-ends coverage (dark blue curve) generally provides better discrimination between methylated positions and FP hits. Improvements related to use of Score 2 calculation scheme are shown by red curve and associated MCC value.

Supplementary Tables

Table S1 List of human rRNA modifications considered in this study
(reference for human 28S rRNA sequence NR_046235 for the position / GeneBank U13369 for the modifications numbering)

RNA	position	modification	RNA	position	modification	RNA	position	modification	RNA	position	modification
5.8S	14	Um14	18S	966	Psi966	28S	1862	Psi1849	28S	3944	Gm3923
5.8S	55	Psi55	18S	1004	Psi1004	28S	1871	Am1858	28S	3959	Psi3938
5.8S	69	Psi69	18S	1031	Am1031	28S	2292	Cm2279	28S	4042	Gm4020
5.8S	75	Gm75	18S	1056	Psi1056	28S	2351	Cm2338	28S	4054	Cm4032
			18S	1081	Psi1081	28S	2363	Am2350	28S	4196	Gm4166
18S	27	Am27	18S	1174	Psi1174	28S	2364	Gm2351	28S	4219	m6A4189
18S	34	Psi34	18S	1238	Psi1238	28S	2365	Cm2352	28S	4227	Um4197
18S	36	Psi36	18S	1244	Psi1244	28S	2401	Am2388	28S	4228	Gm4198
18s	93	Psi93	18S	1248	m1acp3P	28S	2415	Um2402	28S	4293	Psi4263
18S	99	Am99	18S	1272	Cm1272	28S	2422	Cm2409	28S	4296	Psi4266
18S	105	Psi105	18S	1288	Um1288	28S	2424	Gm2411	28S	4299	Psi4269
18S	109	Psi109	18S	1326	Pm1326	28S	2508	Psi2495	28S	4306	Um4276
18S	116	Um116	18S	1328	Gm1328	28S	2787	Am2774	28S	4312	Psi4282
18S	119	Psi119	18S	1347	Psi1347	28S	2804	Cm2791	28S	4353	Psi4323
18S	121	Um121	18S	1367	Psi1367	28S	2815	Am2802	28S	4361	Psi4331
18S	159	Am159	18S	1383	Am1383	28S	2824	Cm2811	28S	4370	Gm4340
18S	166	Am166	18S	1391	Cm1391	28S	2837	Um2824	28S	4392	Gm4362
18S	172	Um172	18S	1442	Um1442	28S	2861	Cm2848	28S	4403	Psi4373
18S	174	Cm174	18S	1445	Psi1445	28S	2876	Gm2863	28S	4420	Psi4390
18S	210	Psi210	18S	1447	Gm1447	28S	3637	Psi3616	28S	4423	Psi4393
18S	218	Psi218	18S	1490	Gm1490	28S	3639	Psi3618	28S	4431	Psi4401
18S	406	Psi406	18S	1625	Psi1625	28S	3695	Psi3674	28S	4442	Psi4412
18S	428	Um428	18S	1639	m7G1639	28S	3701	Cm3680	28S	4443	m5C4413
18S	436	Gm436	18S	1643	Psi1643	28S	3715	Psi3694	28S	4456	Cm4426
18S	462	Cm462	18S	1668	Um1668	28S	3718	Am3697	28S	4457	Psi4427
18S	468	Am468	18S	1678	Am1678	28S	3724	Am3703	28S	4471	Psi4441
18S	484	Am484	18S	1692	Psi1692	28S	3730	Psi3709	28S	4494	Gm4464
18S	509	Gm509	18S	1703	Cm1703	28S	3734	Psi3713	28S	4498	Um4468
18S	512	Am512	18S	1804	Um1804	28S	3744	Gm3723	28S	4499	Gm4469
18S	517	Cm517	18S	1832	m6A1832	28S	3758	Psi3737	28S	4500	Psi4470
18S	572	Psi572	18S	1850	m62A1850	28S	3760	Am3739	28S	4521	Psi4491
18S	576	Am576	18S	1851	m62A1851	28S	3762	Psi3741	28S	4523	Am4493
18S	590	Am590				28S	3764	Psi3743	28S	4530	m3U4500
18S	601	Gm601	28S	398	Am389	28S	3768	Psi3749	28S	4531	Psi4501
18s	609	Psi609	28S	400	Am391	28S	3770	m5C3761	28S	4536	Cm4506
18S	627	Um627	28S	1316	Gm1303	28S	3782	Am3764	28S	4552	Psi4522
18S	644	Gm644	28S	1322	m1A1309	28S	3785	Gm3771	28S	4571	Am4541
18S	649	Psi649	28S	1326	Am1313	28S	3792	Cm3787	28S	4576	Psi4546
18S	651	Psi651	28S	1340	Cm1327	28S	3808	Pm3797	28S	4579	Psi4549
18S	668	Am668	28S	1522	Gm1509	28S	3822	Psi3801	28S	4590	Am4560
18S	681	Psi681	28S	1524	Am1511	28S	3825	Am3804	28S	4618	Gm4588
18S	683	Gm683	28S	1534	Am1521	28S	3830	Am3809	28S	4620	Um4590
18S	686	Psi686	28S	1536	Psi1523	28S	3841	Cm3820	28S	4623	Gm4593
18S	797	Cm797	28S	1582	Psi1569	28S	3844	Psi3823	28S	4628	Psi4598
18S	799	Um799	28S	1625	Gm1612	28S	3851	Psi3830	28S	4636	Psi4606
18s	801	Psi801	28S	1677	Psi1664	28S	3853	Psi3832	28S	4637	Gm4607
18S	814	Psi814	28S	1683	Psi1670	28S	3867	Am3846	28S	4673	Psi4643
18S	815	Psi815	28S	1744	Psi1731	28S	3869	Cm3848	28S	4689	Psi4659

18S	822	Psi822	28S	1760	Gm1747	28S	3884	Psi3863	28S	4972	Psi4937
18S	863	Psi863	28S	1779	Psi1766	28S	3887	Cm3866	28S	5001	Psi4966
18S	866	Psi866	28S	1782	Psi1769	28S	3899	Gm3878	28S	5010	Psi4975
18S	867	Gm867	28S	1792	Psi1779	28S	3920	Psi3899			
18S	918	Psi918	28S	1860	Psi1847	28S	3925	Um3904			

Table S2 Human RiboMethSeq datasets used for extensive analysis and validation

	Cell type	Name	Raw reads	Ref ENA
1	Human umbilical vein endothelial cells (HUVEC)	**Sample1**	22,400,000	PRJEB34951
2	Bone marrow stem cells (BMSC)	**Sample2**	22,600,000	PRJEB34951
3	HeLa	**Sample3**	26,400,000	PRJEB43738
4	HeLa	**HeLa #1**	30,700,000	PRJEB43738
5	HeLa	**HeLa #2**	22,300,000	PRJEB35565
6	HeLa	**HeLa #3**	33,100,000	PRJEB35565
7	HeLa	**HeLa #4**	14,000,000	PRJEB35565
8	HeLa	**HeLa #5**	25,000,000	PRJEB35565
9	HeLa	**HeLa #6**	29,700,000	PRJEB43738
10	HeLa	**HeLa #7**	21,600,000	PRJEB43738
11	HeLa	**HeLa #8**	18,600,000	PRJEB35565
12	Human fibroblasts	**Fibroblasts #1**	21,900,000	PRJEB35565
13	Wharton's Jelly MSC (WJ-MSC)	**Wharton's Jelly MSC#1**	25,100,000	PRJEB35565
14	Human mammary epithelial cells (HME)	**HME #1**	13,200,000	PRJEB35565
15	Human mammary epithelial cells (HME)	**HME #2**	13,300,000	PRJEB35565
16	Human mammary epithelial cells (HME)	**HME #3**	16,000,000	PRJEB35565
17	Human mammary epithelial cells (HME)	**HME #4**	11,300,000	PRJEB35565
18	Human mammary epithelial cells (HME)	**HME #5**	14,600,000	PRJEB35565
19	Human mammary epithelial cells (HME)	**HME #6**	17,600,000	PRJEB35565

Table S3 Comparison of performance for old and new RiboMethSeq scores

	5prime A6 MAX6		*5prime3prime A6 MAX6*		*5prime3prime A2 MEAN2*	
**sample**	**MCC_max6**	**MCC_scoreA6**	**MCC_max6**	**MCC_scoreA6**	**MCC_mean2**	**MCC_scoreA2**
Fibroblasts #1	0.2839	0.2026	0.3605	0.2931	0.4780	0.4407
Sample 1 – HUVEC	0.1546	0.1284	0.2001	0.1540	0.2622	0.2068
Sample 3 – HeLa	0.5802	0.4003	0.7039	0.6273	0.7955	0.7107
HeLa #1	0.6494	0.5632	0.8089	0.8069	0.8249	0.7971
HeLa #2	0.3628	0.2103	0.4175	0.3714	0.5485	0.5058
HeLa #3	0.2340	0.1642	0.2653	0.2134	0.3537	0.3278
HeLa #4	0.3472	0.2266	0.4247	0.3465	0.5669	0.4945
HeLa #5	0.3825	0.2474	0.5084	0.4068	0.6259	0.5523
HeLa #6	0.6788	0.6000	0.8288	0.8618	0.8580	0.8373
HeLa #7	0.6090	0.4598	0.7832	0.7978	0.8398	0.7764
HME #1	0.5295	0.5146	0.7272	0.6960	0.7846	0.7461
HME #2	0.5555	0.4639	0.7344	0.7038	0.7894	0.7360
HME #3	0.5449	0.4880	0.7460	0.7198	0.7855	0.7482
HME #4	0.5453	0.5246	0.7566	0.7166	0.7835	0.7542
HME #5	0.5434	0.4893	0.6921	0.7038	0.7463	0.7341
HME #6	0.5386	0.4951	0.7171	0.7195	0.7776	0.7503
HeLa #8	0.1875	0.1240	0.2192	0.1475	0.2726	0.2337
Sample 2 – BMSC Wharton’s Jelly	0.4021	0.3034	0.5606	0.4658	0.6345	0.5944
MSC#1	0.4509	0.3315	0.6497	0.5664	0.6820	0.6613
**sample**	**FDR_max6**	**FDR_scoreA6**	**FDR_max6**	**FDR_scoreA6**	**FDR_mean2**	**FDR_scoreA2**
Fibroblasts #1	0.7966	0.8315	0.7796	0.8204	0.2857	0.3529
Sample 1 – HUVEC	0.9224	0.8154	0.9045	0.9346	0.8270	0.8985
Sample 3 – HeLa	0.3960	0.4769	0.2745	0.3784	0.0405	0.3070
HeLa #1	0.3053	0.4720	0.0732	0.1124	0.0494	0.1845
HeLa #2	0.6262	0.7368	0.5474	0.5914	0.3288	0.3623
HeLa #3	0.8657	0.9245	0.8620	0.8386	0.5000	0.7377
HeLa #4	0.6381	0.8682	0.4868	0.6939	0.3494	0.2963
HeLa #5	0.5876	0.8579	0.5524	0.5495	0.3529	0.3333
HeLa #6	0.1948	0.5000	0.1400	0.0928	0.0353	0.0690
HeLa #7	0.3258	0.6455	0.0286	0.1633	0.0968	0.2342
HME #1	0.4388	0.4865	0.2752	0.2604	0.1596	0.2083
HME #2	0.4924	0.5635	0.2323	0.2745	0.1163	0.1978
HME #3	0.4961	0.5912	0.2569	0.2982	0.1881	0.2143
HME #4	0.4545	0.4070	0.2632	0.3043	0.1176	0.1915
HME #5	0.5274	0.5175	0.3333	0.2745	0.1868	0.1910
HME #6	0.5147	0.5391	0.2946	0.2111	0.1190	0.2200
HeLa #8	0.9131	0.9331	0.8915	0.8350	0.7868	0.8125
Sample 2 – BMSC Wharton’s Jelly	0.5357	0.7913	0.3000	0.5278	0.2824	0.2740
MSC#1	0.4756	0.7380	0.2237	0.4583	0.2472	0.1831
